# Ecological Stoichiometry of Bumblebee Castes, Sexes, and Age Groups

**DOI:** 10.3389/fphys.2021.696689

**Published:** 2021-10-13

**Authors:** Ronalds Krams, Māris Munkevics, Sergejs Popovs, Linda Dobkeviča, Jonathan Willow, Jorge Contreras Garduño, Tatjana Krama, Indrikis A. Krams

**Affiliations:** ^1^Chair of Plant Health, Estonian University of Life Sciences, Tartu, Estonia; ^2^Department of Biotechnology, Daugavpils University, Daugavpils, Latvia; ^3^Department of Zoology and Animal Ecology, Faculty of Biology, University of Latvia, Riga, Latvia; ^4^Department of Environmental Science, Faculty of Geography and Earth Sciences, University of Latvia, Riga, Latvia; ^5^Escuela Nacional de Estudios Superiores Unidad Morelia, Universidad Nacional Autónoma de México, Morelia, Mexico; ^6^Institute of Ecology and Earth Sciences, University of Tartu, Tartu, Estonia

**Keywords:** bumblebee, *Bombus terrestris*, ecological stoichiometry, the carbon-to-nitrogen ratio, stress, castes, social organization

## Abstract

Ecological stoichiometry is important for revealing how the composition of chemical elements of organisms is influenced by their physiological functions and ecology. In this study, we investigated the elemental body composition of queens, workers, and males of the bumblebee *Bombus terrestris*, an important pollinator throughout Eurasia, North America, and northern Africa. Our results showed that body elemental content differs among *B. terrestris* castes. Young queens and workers had higher body nitrogen concentration than ovipositing queens and males, while castes did not differ significantly in their body carbon concentration. Furthermore, the carbon-to-nitrogen ratio was higher in ovipositing queens and males. We suggest that high body nitrogen concentration and low carbon-to-nitrogen ratio in young queens and workers may be related to their greater amount of flight muscles and flight activities than to their lower stress levels. To disentangle possible effects of stress in the agricultural landscape, further studies are needed to compare the elemental content of bumblebee bodies between natural habitats and areas of high-intensity agriculture.

## Introduction

Ecological stoichiometry focuses on the interactions of organisms with their environments, analyzing how ecological and evolutionary factors shape their bodies’ elemental composition (Sterner and Elser, 2002; Filipiak et al., 2017). Several environmental factors have the potential to influence the elemental composition of organisms, including pollution (De Senerpont Domis et al., 2014; Janssens et al., 2017), ambient temperature (Janssens et al., 2015; Zhang et al., 2016), developmental speed (Trakimas et al., 2019; Krams et al., 2020), and predation risk (Janssens et al., 2015; Krams et al., 2016, 2021; Van Dievel et al., 2016; Zhang et al., 2016). The general stress paradigm states that exposure to stressors generally increases the production of steroid hormones (Hawlena and Schmitz, 2010a; Rinehart and Hawlena, 2020). Stressful conditions generally cause oxidative stress (Janssens and Stoks, 2018), inducing glucogenesis which in turn increases metabolic rate (Slos and Stoks, 2008; Hawlena and Schmitz, 2010a,b; Krams et al., 2013a,b). This increases the demand for carbohydrate-based fuel, containing high levels of carbon (C), and shifting the metabolic balance away from the anabolism that produces nitrogen (N)-rich proteins necessary for producing carbohydrates (Hawlena and Schmitz, 2010a,b; Trakimas et al., 2019). These complex processes generally increase the C/N ratio (Sterner and Elser, 2002; Hawlena and Schmitz, 2010a,b; Van Dievel et al., 2020).

Bumblebees belong to the order Hymenoptera, composed of bees, wasps, and ants. Like other social insects, a bumblebee colony consists of the reproductive queen, mostly sterile workers, and males. Queens are substantially larger than workers, and their development is determined by receiving more food which prolongs their instar stages and increases juvenile hormone levels (Cnaani et al., 1997). The larger fat body [an organ analogous to the liver and adipose tissue in the vertebrates (Schmid-Hempel, 2011)] of queens is a distinctive anatomical trait separating them from workers, and the latter can use the space emptied by their slimmer fat body for filling with nectar (Alford, 1969; Shpigler et al., 2013). Queens establish their own nests, lay a small batch of diploid eggs in spring, collect nectar and pollen, and feed their larvae. As soon as the first workers emerge, queens switch to laying eggs. Bumblebee workers forage for pollen and nectar and tend larvae. Queens and workers also provide nest defense, while mating is the only function of males in the colony (Goulson, 2012). Thus, the division of labor and reproductive investment can affect metabolism, energy budgets, and stress levels in bumblebees (Kelemen et al., 2019). These numerous anatomical, behavioral, developmental, and functional differences can bring different levels of physiological stress may affect the individuals’ stress levels and ecological stoichiometry of their bodies.

In bumblebee nests, individuals may vary in how they are exposed to the environment (Goulson, 2012). Young queens have to find places to overwinter, and they also need to be physically active in foraging, as well as tending to and defending the first workers who are fed solely by queens early in the season. In contrast, ovipositing queens do not forage outside the nest, as this is a task performed by workers. Males provide food only for themselves, their only contribution to the colony being their role in sexual reproduction. This suggests that workers and young queens are individuals most exposed to environmental stressors, such as predators, pesticides, and both intra- and interspecific competition; and they need to rely on their flight muscles the most. These functional differences can affect body elemental composition, especially body N concentration.

The present study investigated whether ovipositing queens, young queens, workers, and males of the buff-tailed bumblebee (*Bombus terrestris*) differ in their body C and N compositions and the C/N ratio, a reliable indicator of physiological stress across taxa (Hawlena and Schmitz, 2010a; Rinehart and Hawlena, 2020). *B. terrestris* is one of the most abundant and widespread bumblebee species in northern Europe with monandrous queens, monandry being associated with increased survival in this species (Baer and Schmid-Hempel, 2005; Goulson, 2012). Bumblebee castes, sexes, and age groups differ in division of labor, quality of food they consume, and longevity, which suggests differences in their stress levels (Schmid-Hempel, 2011; Jedlička et al., 2016). We predicted higher body C concentrations and C/N ratios in workers and young queens if stress is of high importance to bumblebee body composition, and higher body N concentrations and the lower C/N ratios in workers and young queens if body elemental composition depends on behavioral diversity and social roles.

## Materials and Methods

### Insects

We used commercial *B. terrestris* colonies (Koppert Biological Systems, Berkel en Rodenrijs, Netherlands). All colonies were purchased simultaneously and originated from the same breeding line. Colonies were placed in apple orchards in southeast Latvia at the beginning of May 2019. The study area is a mosaic of small private properties containing apple orchards, small vegetable gardens, and meadows interspersed by arable land, pastures, and pine and alder forests.

A total of 31 colonies across 16 apple orchards (ca. 4ha in size) separated by 2–3km were used. At 15 apple orchards, we placed two colonies that were ca. 10m apart from each other. At one apple orchard, we had one bumblebee colony. Each hive was weighed, and the attached plastic container with feeding solution was removed at the moment the colony was allocated to the sampling site. We regularly checked the condition of each colony by observing foraging flights, colony sounds, and fanning behaviors of workers on sunny days. All the colonies were protected against ant attacks by attaching thin durable double-sided tape around the bottom of each hive.

We collected the colonies just as they reached the young queen and male (generative offspring individuals) production phase. Almost all colonies had ovipositing queen, young queens, workers, and males at the beginning of July 2019. Since wings and body hair of old queens become worn and faded over time, we easily distinguished old queens from young queens based on their wing- and hair condition. We prevented both ovipositing and young queens from escaping by using exit holes large enough only for workers and males. We allowed all colony members to enter, but not exit, the colonies 7–9h before collecting the colonies. We collected all colonies from each site and placed hives in a freezer at −80°C.

### Bumblebee Body C and N Content

The percentage of C and N content was measured from the mass of whole bees, using an element analyzer EuroVector EA3000 (Eurovector Srl, Pavia, Italy; Krams et al., 2020, 2021). Samples of C and N concentrations were measured for each ovipositing queen, while in samples of males, workers, and young queens we merged three individual bumblebees for each analyzed sample unit. We had 31 ovipositing queen samples, 28 (*n*=84) young queen samples, 31 (*n*=93) worker samples, and 30 (*n*=90) male samples. C concentration and the C/N ratio are considered important markers of stress (Sterner and Elser, 2002; Hawlena and Schmitz, 2010a,b; Van Dievel et al., 2020). Higher N concentration usually reflects increased allocation to muscle mass, which consists largely of proteins (Fagan et al., 2002). Importantly, several of the most frequently used methods for protein determination are based on analyses of the total N content in samples (Mæhre et al., 2018).

### Statistics

All statistical analyses were performed in R version 4.04 (R Core Team, 2021). We used linear mixed-effects models (Bates et al., 2015) to assess how dry body mass, body C and N concentrations, and the C/N ratio differ among *B. terrestris* castes and sexes. Castes (i.e., workers and queens) and sexes (i.e., males and females) were set as fixed effects, and colony identity, nested within apple orchard identity, was used as a random effect. We used Box–Cox transformation on dry body mass and C/N ratio before fitting respective models to reduce heteroscedasticity. In addition, we fitted linear mixed-effects models to compare dry body mass, body C and N, and the C/N ratio between young and ovipositing queens, using the same random effects as before. Here, we also used Box–Cox transformation on dry body mass, body N, and C/N ratio before fitting respective models to reduce heteroscedasticity. We reported the F-statistic and *p*-value for each linear mixed-effects model and used Satterthwaite’s approximation from lmerTest package (Kuznetsova et al., 2017) to calculate degrees of freedom. In case of significant effect of caste and sex, we performed Tukey’s honest significance test (Tukey HSD) for pairwise comparisons between *B. terrestris* castes and sexes. Differences were considered statistically significant at *p* < 0.05 in all tests.

## Results

**Figure 1:**
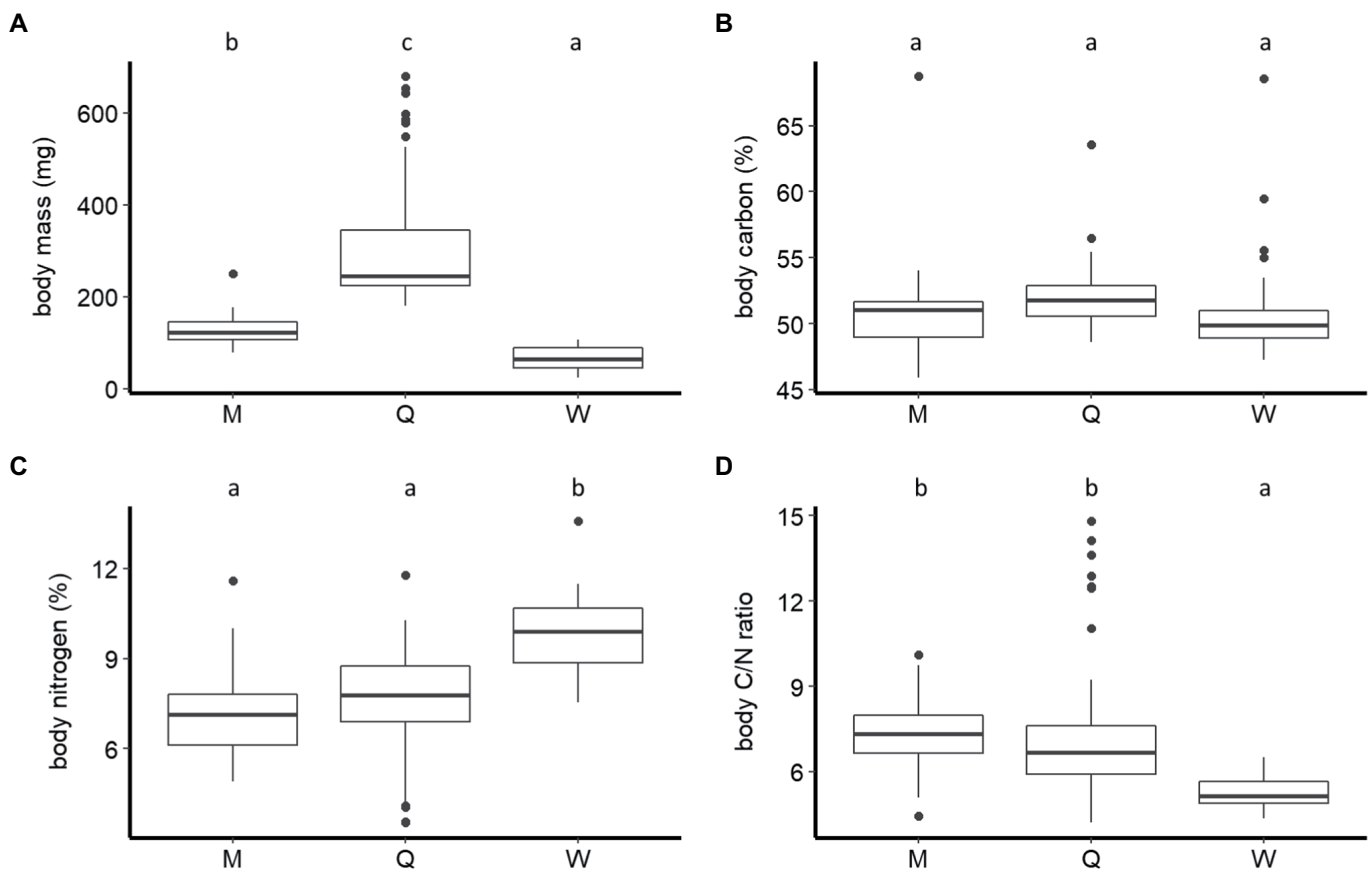
Boxplots of dry body mass **(A)**, carbon, **(B)** nitrogen percentage **(C)**, and carbon-to-nitrogen ratio **(D)** of males (M), queens (Q), and workers (W) of *B. terrestris*. Significantly different means are denoted above each panel by use of compact letter display.

**Figure 2:**
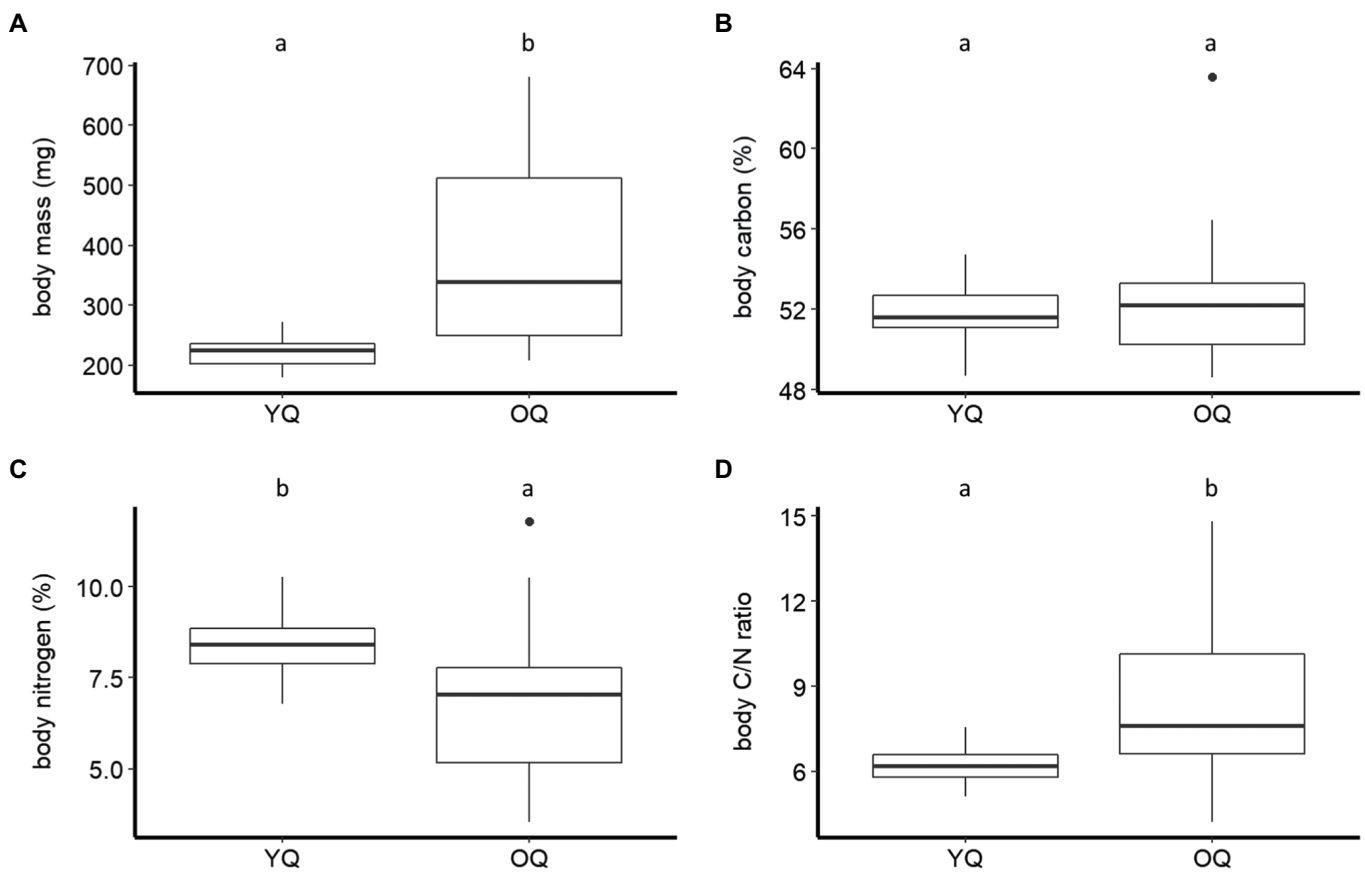
Boxplots of dry body mass **(A)**, carbon, **(B)** nitrogen percentage **(C)**, and carbon-to-nitrogen ratio **(D)** of young queens (YQ) and ovipositing queens (OQ) of *B. terrestris*. Significantly different means are denoted above each panel by use of compact letter display.

The body mass of *B. terrestris* significantly differed between castes and sexes (F_2,110_=185.41, *p* < 0.001). The body mass of queens (307.31±137.31mg, mean±SD) was significantly higher than that of males (128.20±34.60mg). Queens and males were significantly heavier than workers (65.62±25.32mg; Tukey HSD: all *P*s<0.001; Figure 1A). We also found that ovipositing queens (381.84±151.25mg) were significantly heavier than young queens (221.73±26.69mg; F_1,54_=51.45, *p* < 0.001; Figure 2A).

The *B. terrestris* castes and sexes did not differ significantly in their body C concentration (males: 50.95±3.88%, mean±SD; workers: 50.95±4.11%; queens: 52.05±2.33%; F_2,115_=1.61, *p*=0.2; Figure 1B). Queens of different ages also did not differ significantly in their body C (ovipositing queens: 52.30±2.85%, mean±SD; young queens: 51.77±1.52%; F_1,54_=1.23, *p*=0.27; Figure 2B).

Castes did, however, differ significantly in body N concentration (F_2,111_=27.04, *p* < 0.001). Body N concentration was significantly higher in workers (9.76±1.37%, mean±SD) than in queens (7.57±1.79%) and males (7.1±1.45%; both *P*s<0.001). Males and queens did not differ significantly (*p*=0.47) in their body N (Figure 1C). Body N concentration also differed significantly between queen age groups (F_1,54_=12.42, *p* < 0.001). It was higher in young queens (8.4±0.84%), than in ovipositing queens (6.85±2.08%; Figure 2C).

Body C/N ratio was significantly different between castes (F_2,111_=35.36, *p* < 0.001). The body C/N ratio did not differ statistically between queens (7.41±2.43, mean±SD) and males (7.41±1.3; *p*=0.22; Figure 1D), but it was significantly higher than the body C/N of workers (5.29±0.62; *P*s<0.01; Figure 1D). Body C/N was also statistically different between queens of different ages (F_1,54_=13.64, *p* < 0.001). Ovipositing queens (8.44±2.91) had significantly higher C/N than young queens (6.22±0.62; Figure 2D).

## Discussion

Elemental body content of insects is largely formed during larval and pupal development (Scotto-Lomassese et al., 2003). Therefore, the ecological stoichiometry of bumblebees is linked with either stress levels experienced during their larval and pupal development, or possible differences with the functional roles of bumblebee castes during adulthood, both of which affect C and N concentrations in their bodies.

Females, especially queens, receive extra food during their larval development, which results in their high body N concentration. Nitrogen is an element associated with an investment in protein production (Gordon et al., 2013), which may result in greater muscle mass, behavioral diversity, and rapid behavioral responses under predation risk (Krams et al., 2016). Queens need highly developed flight muscles to have a higher speed at take-off and better maneuverability while escaping predators (Almbro and Kullberg, 2012). At the beginning of their reproductive season, each bumblebee queen must find a safe place to establish a colony. Then, they must collect food and provide thermoregulation and antipredator protection to their offspring, until the first generation of workers emerges and takes over foraging and antipredator protection functions. After that point, ovipositing queens will not leave their nests to collect nectar and pollen.

Bumblebees are known to have colony-level thermoregulation to survive periods of cold or hot weather, and this is provided by either fanning behavior or raising body temperature. While colony-level thermoregulation is important for rearing the young, bumblebees also need to individually warm up to fly and forage during the early hours of the day, or in colder climates during the whole day (Bujok et al., 2002; Seeley et al., 2003). The temperature of bumblebee flight muscles needs to reach at least 30°C (Goller and Esch, 1990). This can be reached by flight muscle shivering. This process may be surprisingly fast, and the increased temperature is often restricted to the thorax, the mid-section of the body that is packed with flight muscles. Generation of heat can also be achieved without shivering of fibrillar muscles, which contract when they receive an action potential (Esch et al., 1991). A flight muscle receives up to 40 action potentials per second, and this substantially increases tension yet results in little motion (Heinrich, 1975). These functions are normally performed by workers and young queens, whereas ovipositing queens provide thermoregulation only at the early stages of the reproductive cycle. Overall, this may explain high body N concentration and the low C/N ratio of workers and young queens. Males are neither involved in providing the thermoregulatory function of their colonies, nor in collecting food for the developing larvae. The lack of these functions may affect the amount of muscles in males and increase their C/N ratio. The relatively low body N concentration, and subsequently lower C/N ratio, of ovipositing females may be explained by their larger fat body, and a sizable fraction of the body of a mated female is made up of developing eggs and supportive tissue (Anderson et al., 1979; Burggren et al., 2017). This leads to higher body mass in ovipositing queens compared to that of young queens.

We suggest that similar body C concentrations across castes and sexes likely indicate low stress levels in the studied bumblebees. If environmental stress were important, we would find higher C/N ratios than found in this study in workers and young queens compared to ovipositing queens and males due to decreased body N concentration. According to the general stress paradigm, the decrease in body N is expected because higher stress levels would break muscle tissues into carbohydrate-based fuel (Hawlena and Schmitz, 2010a,b; Rinehart and Hawlena, 2020). However, body N concentrations were the highest in workers and young queens, which rely on functional flight muscles more than ovipositing queens and males. On the other hand, it has been shown that heightened stress levels may affect reproductive responses in insects (Adamo and McKee, 2017) by decreasing body mass and potentially increasing body N concentration and reducing the C/N ratio. It has been recently shown that fruit flies reared together with predators were found to be somewhat smaller (Krams et al., 2016, 2020). This made them more agile in negative geotaxis tests and improved their survival under spider predation because of larger relative muscle mass associated with a higher body N concentrations and reduced C/N ratios. Future research is needed to test whether stressed bumblebees are smaller compared to non-stressed individuals.

Our results on the relatively high C/N ratio of males and ovipositing queens may likely be explained by their low involvement in regular physical activities, rather than differences in stress. We suggest that bumblebees’ stress levels may have been low because the study site was located in a landscape of relatively low-intensity agriculture and low intensity of competition/disturbance from other species, such as ants. Therefore, future research is needed to compare between-population (i.e., ecological factors) and within-population (i.e., castes, sexes, and queen age groups) differences in body sizes and the ecological stoichiometry of *B. terrestris* in landscapes of both high- and low-intensity agriculture, in order to detect baseline concentrations of C and N and better understand the roles of social organization and environmental stress on body elemental content. We also suggest measurements of flight muscle mass to better understand variations of N between castes and sexes of bumblebees. Finally, measurements of reactive oxidative species (ROS) and reactive nitrogen species (RNS) and the tissue’s protective systems such as mitochondrial and cytosolic isoforms of superoxide dismutase, catalase, and glutathione peroxidase enzymes, and several direct scavengers of ROS and RNS, including glutathione, vitamin E, and ascorbate, must be done in the future (Sharifi-Rad et al., 2020). This approach is critical because oxidative stress is not caused solely by excessive production of ROS and RNS but by the imbalance between reactive species caused by stress and protective antioxidative systems (Monaghan et al., 2009; Sharifi-Rad et al., 2020).

## Data Availability Statement

The raw data supporting the conclusions of this article will be made available by the authors, without undue reservation.

## Author Contributions

RK, TK, JCG, and IK designed research. RK, SP, MM, TK, and IK performed research. RK, TK, SP, and LD analyzed samples. RK, MM, JCG, LD, and IK analyzed data. RK, JW, and IK wrote the paper. All authors contributed to the article and approved the submitted version.

## Funding

Funding was provided by the Latvian Council of Science (grants lzp-2018/1-0393, lzp-2018/2-0057, and lzp-2020/2-0271) and the Estonian Research Council (grant PUT1223).

## Conflict of Interest

The authors declare that the research was conducted in the absence of any commercial or financial relationships that could be construed as a potential conflict of interest.

## Publisher’s Note

All claims expressed in this article are solely those of the authors and do not necessarily represent those of their affiliated organizations, or those of the publisher, the editors and the reviewers. Any product that may be evaluated in this article, or claim that may be made by its manufacturer, is not guaranteed or endorsed by the publisher.
